# Dolichens: An Information System on the Lichens of the Dolomites

**DOI:** 10.3390/jof11090624

**Published:** 2025-08-26

**Authors:** Luana Francesconi, Matteo Conti, Stefano Martellos, Luca Di Nuzzo, Gabriele Gheza, Pier Luigi Nimis, Chiara Pistocchi, Juri Nascimbene

**Affiliations:** 1 BIOME Lab, Department of Biological, Geological and Environmental Sciences, Alma Mater Studiorum University of Bologna, Via Irnerio 42, I-40126 Bologna, Italy; luana.francesconi3@unibo.it (L.F.); luca.dinuzzo2@unibo.it (L.D.N.); gabriele.gheza@unibo.it (G.G.); chiara.pistocchi2@unibo.it (C.P.); juri.nascimbene@unibo.it (J.N.); 2 Centro Interuniversitario per le Biodiversità Vegetale Big Data—PLANT DATA, Department of Biological, Geo-logical and Environmental Sciences, Alma Mater Studiorum University of Bologna, I-40126 Bologna, Italy; matteo.conti26@unibo.it; 3 Department of Life Sciences, University of Trieste, Via Giorgieri 10, I-34127 Trieste, Italy; nimis@units.it

**Keywords:** Alps, biodiversity informatics, Darwin Core, data mobilization, open data, web-GIS

## Abstract

Lichens, despite their key role as environmental indicators and their ecological importance, remain underrepresented in conservation policies, largely due to fragmented exploration of several areas, as well as limited availability of data in digital platforms. The UNESCO World Heritage area of the Dolomites (N Italy) is well-investigated as far as lichen diversity is concerned, with a long history of lichenological exploration since the 19th century. However, the relevant amount of data produced by these efforts was scattered and often not accessible in digital format, thus hindering data accessibility and usability. In this paper, we present Dolichens, a novel web platform designed to aggregate data about lichen diversity in the Dolomites. The platform aims at making available a comprehensive resource to support research, monitoring, and conservation of lichen diversity in the Dolomites, while ensuring data interoperability with the most relevant global repositories.

## 1. Introduction

Lichens are a complex symbiotic association among a heterotrophic fungus, autotrophic photosynthetic partners (green algae and/or cyanobacteria), and several other microorganisms [1,2]. They are widespread across most of the world’s terrestrial ecosystems [3], and, thanks to their tolerance to desiccation [4], radiation [5], and extreme temperatures [6], they often dominate habitats with conditions too harsh for permanent survival of vascular plant populations [7], e.g., arctic tundra, boreal forest floors [8], and soils and rocks surfaces in alpine areas [9].

Lichens play several key roles in ecosystems [10]: they are involved in carbon and nitrogen fixation [11], rock weathering, and soil formation and stabilization [12]. Furthermore, they provide habitat, food, and nesting material to several organisms [13,14].

Lichens are also sensitive to environmental changes, such as habitat fragmentation and degradation [15], air pollution [16], and climate change [17]. They are often used as bioindicators of air quality [18] and forest health [19].

However, lichens are often underrepresented in conservation policies and management guidelines [20], even if some efforts have been carried out in this direction, at least in some countries such as Italy [21,22]. This situation is at least partly related to a scarcity of reliable and expert-validated occurrence data, limiting proper conservation and distributional assessments [23,24]. Currently, only a few global expert-curated resources on lichens are currently available (e.g., the Consortium of Lichen Herbaria [25] and the LIAS [26]). General biodiversity repositories like GBIF [27] and iDigBio [28], while useful to supplement specialized databases, require careful quality control checks before the use of the data they aggregate [29].

As far as lichens are concerned, Italy is among the most extensively studied countries [30], and, at the country level, data are aggregated into ITALIC, the Information System on Italian Lichens [31]. Up to date, ITALIC organizes information for more than 3300 taxa, including nomenclature, systematics, ecology, morpho-functional traits, images, and identification keys.

Even in the case of Italy, there is still a relevant knowledge gap, due to the scarcity of curated and reliable occurrence records available in digital format. In order to fill this gap, in 2023, the aggregation of occurrence data from modern herbaria was started [31]. Unfortunately, occurrence data are still fragmented across different resources (including paper-printed scientific journal articles), unevenly distributed across the country, and often not available online, hindering robust distribution modeling [32,33] to support conservation assessments and policies [23]. However, mobilization and aggregation of these data on a large spatial scale (nation- or continent-wide) can require a huge amount of time and expert effort, which are hardly feasible without proper planning, funding, and multi-center collaboration. Therefore, focusing on relatively small, well-defined geographical areas offers a pragmatic approach for addressing the issue of data scarcity and aligns well with conservation implementation practices [34].

In this perspective, mountain areas are particularly interesting, as they host several taxa of conservation concern [35], as well as diversity hotspots [36]. In the Southern European Alps (NE Italy), the Dolomites are an emblematic mountain region, being included in the UNESCO World Heritage list since 2009 [37] and hosting several protected areas. The Dolomites boast a long tradition of lichenological exploration, which started in the 19th century with Ferdinand Arnold (1828–1901) and Ernst Kernstock (1852–1900) and continued until recent times with several sampling campaigns, often supported and/or commissioned by Natural Parks [38].

The Dolichens web platform was developed as a repository for lichen occurrence data from the Dolomites, aimed at supporting research and conservation planning. It aggregates data that were previously not available online and were scattered among several data sources, digital or paper printed. The platform was developed by extending the ITALIC codebase [31], while adding new features specifically designed to retrieve and manage occurrence data.

The main objectives of this research are as follows:

1. The creation of a comprehensive, regularly updated database of the lichens occurring in the Dolomites.
2. The development of a website for accessing and downloading the data.
3. The interoperability of the aggregated data with global biodiversity repositories, such as the GBIF.
4. The reuse of ITALIC codebase for the creation of information systems focused on different geographic circumscriptions (from local to global).

## 2. Materials and Methods

### 2.1. Study Area

**Figure 1:**
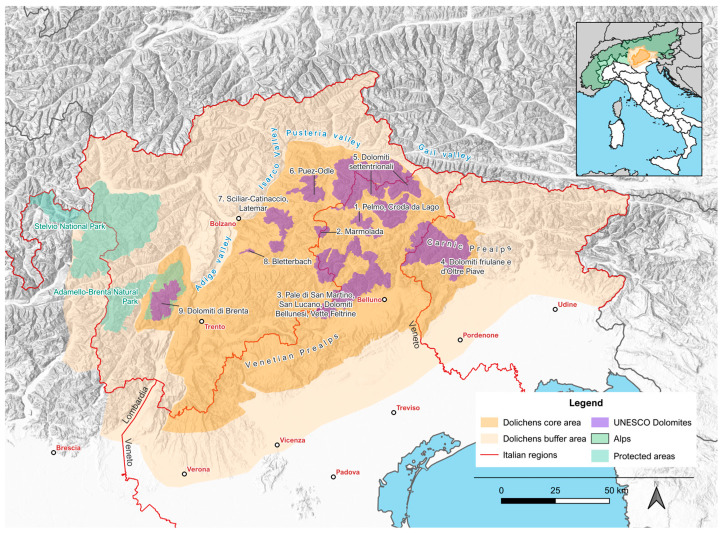
Study area of the project, divided into the core area (dark orange) and the buffer area (light orange).

The project encompasses two areas: a core area, limited to the actual Dolomites as defined by UNESCO [37], and a buffer area around it (Figure 1).

The core area embraces all the main Italian dolomitic massifs. Spanning 11,735 km^2^, it is located in NE Italy, encompassing portions of the administrative regions Trentino-Alto Adige, Veneto, and Friuli Venezia Giulia. It is located to the south of the Pusteria and Gail valleys and to the east of the Adige and Isarco valleys. It includes the Brenta Dolomites and the Friulan Alps on the west and reaches south to the Venetian and Carnic Prealps, also including the “Little Dolomites” [39]. 

The buffer area surrounds the core area. On the north and east, its boundary follows the national border, on the west it encloses the Stelvio National Park and the Adamello-Brenta Natural Park, while on the south it runs along the Po Valley, up to the border of the municipality of Verona.

### 2.2. Data Sources

Occurrence data were obtained from two main sources: herbaria and the literature. As far as herbaria are concerned, data were retrieved from modern Italian herbaria aggregated in ITALIC [31], historical herbaria, Natural Park collections, and datasets accessible through the Global Biodiversity Information Facility (GBIF) [27] and Consortium of Lichen Herbaria database [25].

Literature data were gathered from checklists, vegetation surveys, monitoring reports, taxonomic revisions, and distributional studies published since the 19th century [38]. Additionally, occurrence records from recent sampling campaigns, grey literature (e.g., bachelor’s and master’s theses, technical reports), and unpublished data from the authors of this manuscript were included as well.

Data were collected primarily for the core area. Data from the buffer zone were collected only when they were included in the same data sources containing data for the core area.

Furthermore, to address the underrepresentation of common and easily identifiable species, an iNaturalist project was created (https://www.inaturalist.org/projects/dolichens-project-the-lichen-biota-of-the-dolomites accessed on 18 August 2025).

The taxonomic backbone of the system, which includes accepted names, basionyms, and synonyms of taxa occurring in the study area, is the Checklist of the Lichens of Italy [30], available online in ITALIC [31].

### 2.3. Data Aggregation and Interoperability

For each occurrence record, when possible, the following minimal set of information was retrieved: name of the taxon in the original source, locality, coordinates, collection date or year, substrate, habitat, altitude, collector, and identifier.

To support standardization, aggregation, and interoperability of data, we developed a processing pipeline with multiple normalization and validation steps:

- All occurrence records were aligned to the Darwin Core metadata standard [40,41]. Since the same record may appear in multiple datasets, duplicates have been automatically removed. Potential duplicates (records with the same scientific name, locality, and collector but discrepancies in other metadata) have been flagged for manual revision.
- Georeferenced occurrences have been reviewed and, if necessary, corrected based on the description of the collection locality. When geographical coordinates of the collection locality were missing, the records were georeferenced a posteriori using Google Maps, Google Earth, and regional GIS maps, following the best practices proposed by Chapman and Wieczorek [42].
- For georeferenced occurrence records, supplementary geographical data, including elevation, administrative region, drainage basin, and SOIUSA (Suddivisione Orografica Internazionale Unificata del Sistema Alpino [43]), were derived from Digital Elevation Model (DEM) maps and administrative boundary shapefiles. In this phase, it was also automatically checked whether an occurrence was collected within a protected area (national area design and Natura2000 network).
- Data were labeled as belonging to one of four categories: floristic surveys (occurrence data from checklists, floras, taxonomic revisions), vegetation plots (occurrences derived from co-occurrence data gathered through vegetation surveys and monitoring), observations (field observations and photos), or herbarium specimens.

### 2.4. Taxonomic Alignment

Each scientific name was matched against the Checklist of the Lichens of Italy [30] by means of the tools available in ITALIC [31], to verify whether a name is accepted, a synonym, or an unmatched entry. The original name, as reported by the source, was always retained alongside the accepted name.

After the nomenclatural harmonization, records of taxa that have never been reported for the study area were automatically identified and flagged. After a manual review process, each of these observations was either verified and reported as the first occurrence of the taxon in the area, or marked as dubious, thus stimulating further investigation.

### 2.5. The Dolichens Information System

**Figure 2:**
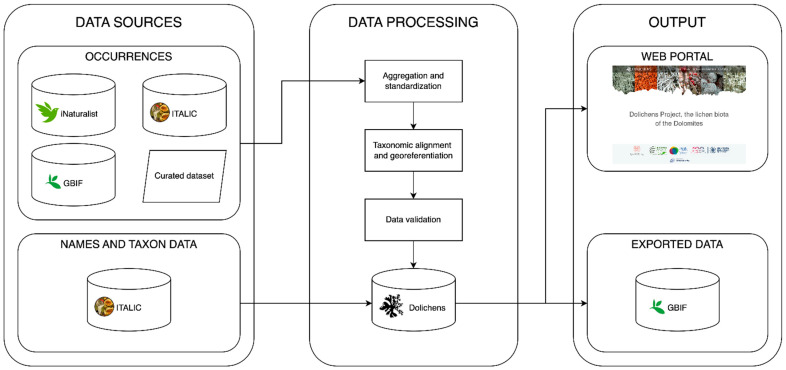
Data flow through the Dolichens infrastructure. Occurrence data are taken from multiple sources and pass through different processing steps before being stored in the database. Scientific names along with taxon-related data are sourced from ITALIC. All the aggregated data are made available through a website, while new occurrences are regularly shared with the GBIF.

The information system runs on an Apache web server. The backend is written in PHP 8.2, and the data are organized in a MariaDB 11.0 database. Data sourced from ITALIC are periodically harvested using the ITALIC public Restful APIs (https://italic.units.it/?procedure=api accessed on 18 August 2025). The whole system operates as schematized in Figure 2.

On the client side, the application is built with JavaScript using the jQuery library.

All the data in the system can be visualized and downloaded from the website https://italic.units.it/dolichens (accessed on 18 August 2025), which is described in the results section; additionally, new records are periodically published in the GBIF.

## 3. Results

The system aggregates 78,424 occurrence records for 2087 infrageneric taxa (as of June 2025), i.e., 73.7% of the taxa reported from Italy [30] and 65.8% from the Alps [44]. 

**Figure 3:**
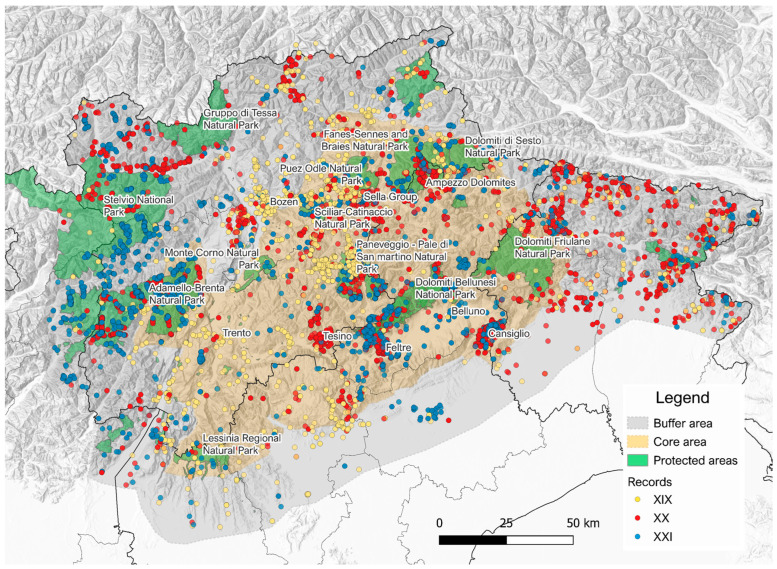
Distribution map of occurrence records in the Dolichens database (created using QGIS software 3.34.6).

Georeferenced records are 98% of the total, and their distribution is shown in Figure 3.

**Figure 4:**
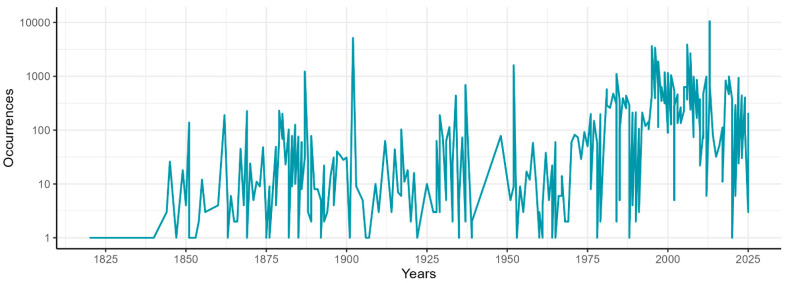
Temporal distribution of the occurrences in the database. The number of occurrences is displayed in logarithmic scale.

**Figure 5:**
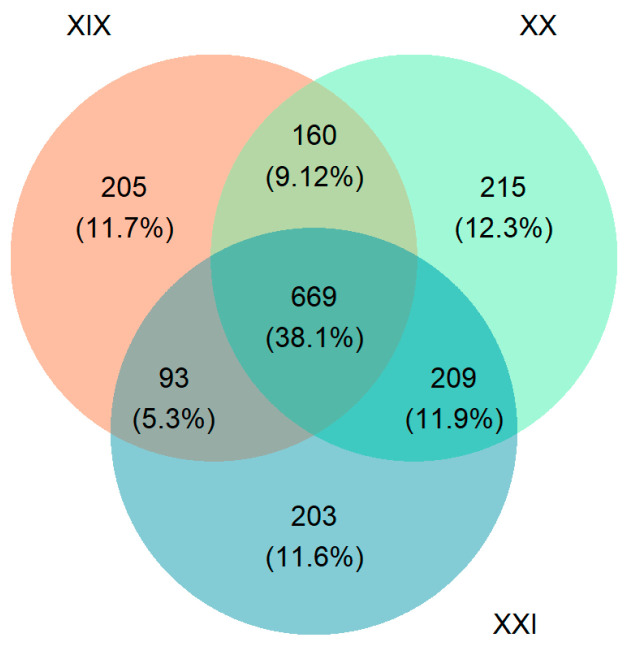
Number of lichen taxa recorded in the three centuries and their overlapping.

Occurrence records describe two centuries of exploration in the Dolomites, spanning from 1822 to 2025 (Figure 4). The distribution of the reports of different taxa since the 19th century is shown in Figure 5.

Half of the records originate from vegetation surveys (38,752, 49.9%), 25.3% (19,641) from floristic surveys and taxonomic revisions, 24.5% (19,011) from herbaria, and 0.3% (249) from field observations. The majority of floristic occurrence records derives from literature sources (18,123), while only a small amount derives from unpublished sources and grey literature (1518). In contrast, records obtained from vegetation surveys mostly come from unpublished sources, grey literature (21,145), and the literature (17,607). As far as herbarium specimens are concerned, 13,450 records were already aggregated in ITALIC, 3095 were obtained from the GBIF [45], while 2832 were obtained from other herbaria [38].

Forty-three percent of the occurrences are located within a protected area instituted at the national level (e.g., Natural or National Parks and Reserves), whereas 16% occur in other areas included in the Natura 2000 Network.

A web portal, available at https://italic.units.it/dolichens (accessed on 18 August 2025), was built to allow users to explore and retrieve the aggregated data. The portal includes two main components:

1. An information section detailing the project, the study area, and the aggregated data;
2. A data query and retrieval module with three different interfaces, each designed to address distinct research needs.

**Figure 6:**
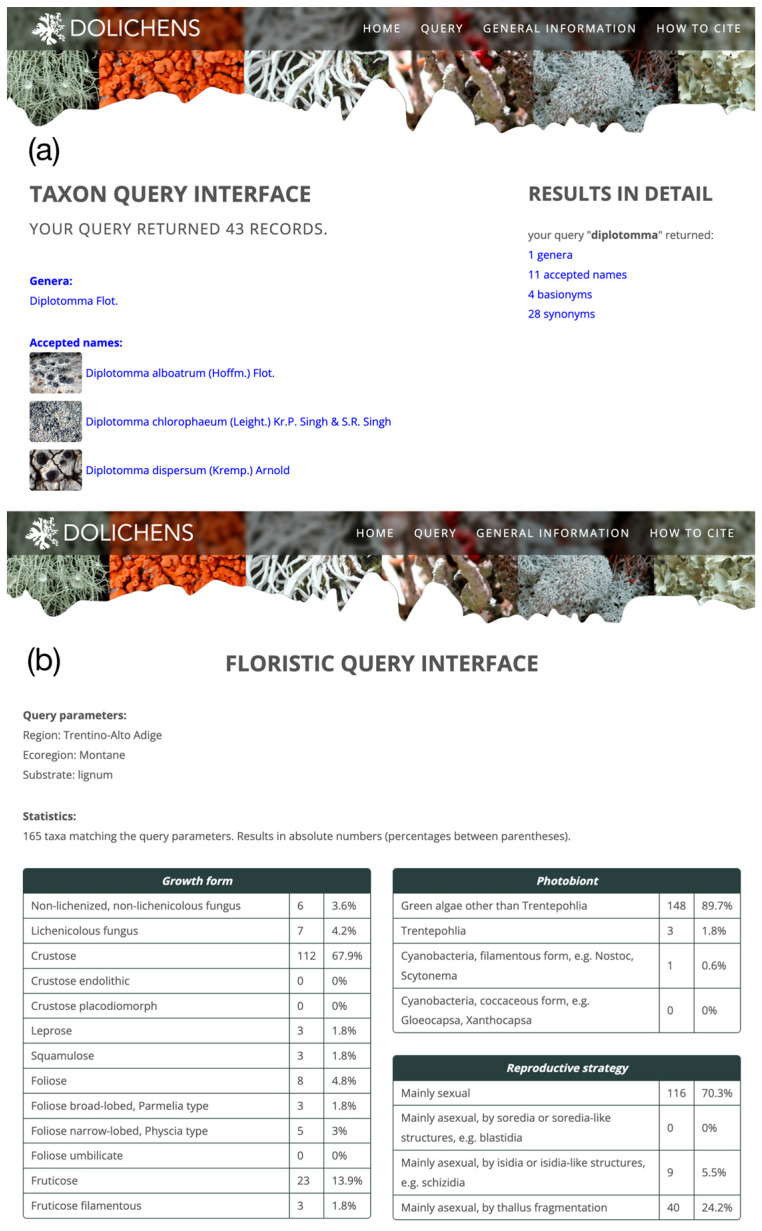
(**a**) Result of the taxon query for “Diplotomma”, the page shows the accepted names along with basionyms and synonyms containing the searched term. (**b**) Result of floristic query showing the list of taxa and statistics about the species occurring in the montane ecoregion of Trentino Alto Adige growing on wood.

The taxon (Figure 6a) and the floristic (Figure 6b) query interfaces derive from ITALIC. The former allows users to search for taxa by scientific names, while the latter generates lists of taxa based on user-defined environmental parameters (for details, see [31]).

**Figure 7:**
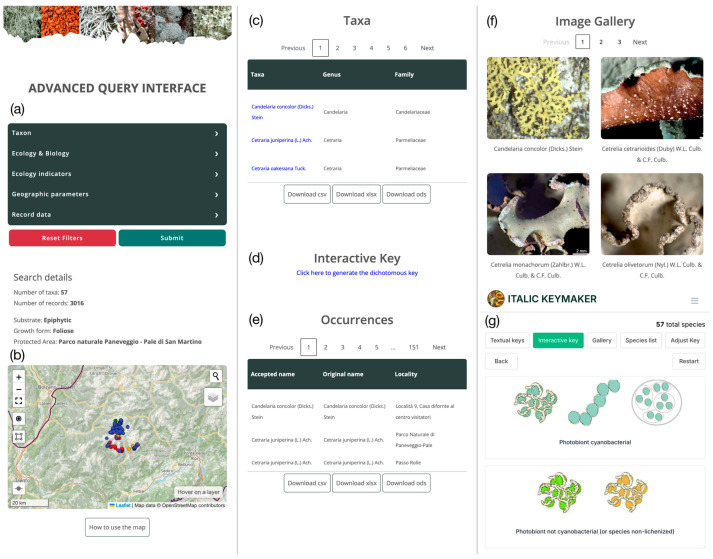
Advanced query interface showing the results of a query for epiphytic foliose lichens in the area of the Paneveggio–Pale di San Martino Natural Park. (**a**) Menu with selectable filters, (**b**) web-gis map (dots color code: blue: herbaria; green: floristic surveys; red: vegetation surveys; orange: observations), (**c**) table of taxa, (**d**) link to dichotomous key, (**e**) table of occurrences, (**f**) image gallery, (**g**) dichotomous identification key interface.

The advanced query interface (Figure 7), on the contrary, was developed in the framework of this study.

The advanced query interface allows users to perform complex queries selecting among a wide array of taxon-related (e.g., systematics, ecology, biology) and occurrence-related filters (e.g., geographic parameters, record data). Additionally, users can select an area on an interactive map as a further geographic filter. The result of a query (Figure 7) is as follows:

1. An interactive map of occurrence records. By clicking on an occurrence, it is possible to display its details, such as the scientific name, the source of the datum, and the locality of collection (with coordinates and their uncertainty).
2. A table of taxa, along with their morpho-functional traits and ecological indicator values.
3. A customized dichotomous identification key to the taxa matching the query, which is derived from the general key to the lichens of Italy.
4. A table of occurrences, with verbatim name, accepted name, locality, coordinates (with uncertainty), and source of the record.
5. A gallery of images of the taxa matching the query.

**Figure 8:**
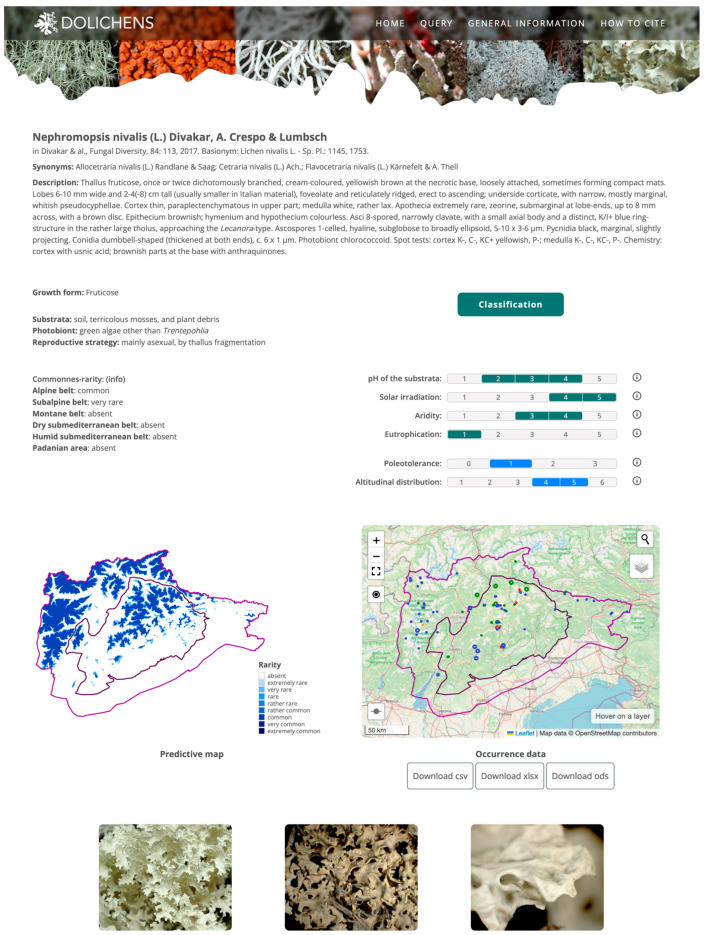
Taxon page of *Nephromopsis nivalis* (L.) Divakar, A. Crespo & Lumbsch. Lines in the maps delimitate the core Dolomites area (black line) and the buffer zone (violet line). Dots color code: blue: herbaria; green: floristic surveys; red: vegetation surveys; orange: observations.

All taxa are linked to their taxon pages (Figure 8). A taxon page provides the following information: (a) accepted name and basionym (if applicable), with a reference to the protologue; (b) a list of synonyms; (c) a set of morpho-functional traits and ecological indicator values; (d) the position in the current phylogenetic tree; (e) commonness–rarity status in each ecoregion of the study area; (f) a map showing the distribution of occurrence records; (g) images with their metadata.

## 4. Discussion

The Dolichens information system was developed to be a comprehensive repository of lichen data for the Dolomites and the Italian Eastern Alps. It aggregates data that were previously available from separate sources or not available in the digital domain. Hence, it offers a unique, centralized access point to retrieve expert-curated information useful for researchers, conservation practitioners, and policymakers. The aggregated data offer a comprehensive overview of the lichen diversity of the Dolomites, which encompasses more than 70% of the species currently known to occur in Italy [30] and more than 60% of those listed for the Alps [44].

As far as the technical features are concerned, the Dolichens information system builds upon the codebase and resources of ITALIC [31], reusing and integrating them to create a new system focused on a specific, restricted geographical area. This approach allows, on one side, to easily integrate the features developed for ITALIC, as well as those which will be developed in the future, in any derived system such as Dolichens. On the other hand, the codebase of ITALIC can be easily fine-tuned and implemented, allowing for the development of specific features for any derived system, as is the case with the new advanced query interface of Dolichens (Figure 7). This interface, developed in the framework of this research, supports the combination of several taxonomical, ecological, and morphological parameters in complex queries. While this advanced feature is mostly devoted to researchers, it could be easily used by anybody thanks to its graphic interface.

As far as data are concerned, the Dolichens project prioritizes quality over quantity. Before aggregation, data are curated through a series of quality control and interoperability checks (see data and methods for details), which aim at aggregating high-quality, verifiable data only, avoiding errors which can occur in botanical databases (see as example [46]). For this reason, at the moment, occurrence records by amateurs and citizen scientists are excluded, due to concerns regarding their reliability [47]. In biodiversity research, occurrence data produced by citizen scientists are often questioned as far as the risk of misidentification is concerned [48,49]. Since lichen identification is a challenging task, even to experts, correct identification rates range from 5% up to ca. 85%, depending on the features that are required for the identification [50,51,52]. Thus, observations collected by amateurs and citizen scientists will be taken into consideration only when coming from initiatives where proper, solid protocols for quality control and verification will be applied.

Some records from the scientific literature, as well as from herbaria, particularly historical ones, can sometimes be misidentified (see as examples [53,54]), and therefore, when spotted, they have been flagged in the system as ‘dubious’. This is, as an example, the case of *Ramalina lacera* (With.) J.R. Laundon and *Ramalina pusilla* Duby, reported by Cengia Sambo [55]. The two taxa are known to be adapted to the Mediterranean climate, with a distribution in the country limited to the peninsula, ranging from Tuscany to Sicily [30]. Thus, the two taxa can hardly be present in the Dolomites, where the climatic conditions are completely different.

Regarding the spatial distribution of the data, a heterogeneous coverage of the Dolomites can be evidenced, with data-rich areas interspersed with under-explored areas (Figure 3). Among the latter, there are the protected areas of the Piccole Dolomiti and Friulian Dolomites, as well as areas such as Valsugana and Lagorai mountain chain. This can be a useful indication to direct future exploration efforts aimed at improving the lichenological investigation of the area.

Despite the disparity in the number of records across the three centuries covered by the Dolichens database, the number of taxa reported in each century is relatively consistent: 1174 in the 21st, 1253 in the 20th, and 1127 in the 19th century. However, comparing the three centuries, on one hand, large overlaps in the taxonomic lists emerge, while on the other, pools of species (about 200 for each period) are peculiar to each century. The reasons for this pattern may be diverse, and their detailed investigation goes beyond the scope of this work. It is likely that there could have been local extinctions, as probably happened in the case of *Ramalina dilacerata* (Hoffm.) Hoffm., which was only reported in the 19th century by Arnold [56] and never found again later in the area. However, these differences may also be attributed to cases of under-exploration, especially as far as many poorly detectable small lichens are concerned, which can easily escape observation. Furthermore, sampling approaches have changed significantly over time. Historically, researchers often relied on preferential sampling, selecting sites or substrates that appeared promising or species-rich. Since the 1980s–1990s, however, there has been a strong push toward random and systematic sampling protocols, particularly in large-scale monitoring programs. These methodological shifts may have influenced reported biodiversity patterns, making comparisons across different periods more complex. In general, despite the limitations related to a non-homogeneous dataset that is not based on systematic sampling, the temporal analysis of the lichen biota included in Dolichens will contribute to clarifying the dynamics of these organisms, possibly also in a perspective of global changes [57].

Aiming at increasing lichen data availability and interoperability, occurrence records that were not publicly available are being periodically published on the GBIF. To date, the Dolichens dataset aggregated in the GBIF contains 56,251 records of 1719 infrageneric taxa [38]. Its publication provided an increase of 392% in lichen occurrences, as well as an increase of 20% in the number of taxa for the Dolomites and Italian Eastern Alps area in the GBIF.

In conclusion, the Dolichens database offers researchers a freely available and reliable source of occurrence data that can serve as a solid basis for challenging open questions in the taxonomy, ecology, and biogeography of the lichen biota. Indeed, the system can be used as a support to multiple approaches, as in the case of Species Distribution Modelling, gradient analyses, temporal analyses, taxonomical revisions, and circumscription of poorly known taxa that were found only in their type locality. Furthermore, the Dolichens system is expected to pave the ground for the development of similar systems at different scales, strongly impacting the accessibility and interoperability of biodiversity data, not limited to lichen diversity alone.

## Data Availability

The data presented in this study are openly available in Dolichens at https://italic.units.it/dolichens/.
